# Supplementary material for: Visuomotor mismatch EEG responses over occipital cortex of freely moving human subjects
Source: eLife. 2026 Aug 20;14:RP108941. doi: 10.7554/eLife.108941 (PMC13493123; doi:10.7554/eLife.108941)
Supplement: Supplementary file 2 [file elife-108941-supp1.docx]

|  | **Comparison** | **Test** | **N_1_** | **N_2_** | **Unit** | **P-value** |
| --- | --- | --- | --- | --- | --- | --- |
| **Figure 1E** | Visual responses during sitting | - | 29 | - | Participants | - |
| **Figure 1F** | Visual responses during walking | - | 29 | - | Participants | - |
| **Figure 1F**  **Inset** | Visual responses during sitting vs walking | Bootstrap | 29 | 29 | Participants | Black bar:  p < 0.05  Gray bar:  p > 0.05 |
| **Figure 2E** | Visuomotor mismatch responses | - | 75 | - | Participants | - |
| **Figure 2F** | Playback halt responses | - | 42 | - | Participants | - |
| **Figure 2G** | Visuomotor mismatch vs playback halt responses | Bootstrap | 75 | 42 | Participants | p < 0.001 |
| **Figure 2H** | Closed loop vs open loop visual flow re-onset responses | Bootstrap | 75 | 42 | Participants | p = 0.4531 |
| **Figure 2I** | Walking speed during visuomotor mismatch vs baseline walking speed | Bootstrap | 72 | - | Participants | Gray bar:  p > 0.05 |
| **Figure 3C** | Visuomotor mismatch responses vs 0 on Fp1 | Bootstrap | 51 | - | Participants | p < 0.001 |
|  | Visuomotor mismatch responses vs 0 on Fp2 | Bootstrap | 51 | - | Participants | p < 0.001 |
|  | Visuomotor mismatch responses vs 0 on C3 | Bootstrap | 27 | - | Participants | p = 0.1758 |
|  | Visuomotor mismatch responses vs 0 on C4 | Bootstrap | 30 | - | Participants | p = 0.2193 |
|  | Visuomotor mismatch responses vs 0 on P3 | Bootstrap | 32 | - | Participants | p < 0.001 |
|  | Visuomotor mismatch responses vs 0 on P4 | Bootstrap | 33 | - | Participants | p = 0.0065 |
|  | Visuomotor mismatch responses vs 0 on O1 | Bootstrap | 66 | - | Participants | p < 0.001 |
|  | Visuomotor mismatch responses vs 0 on O2 | Bootstrap | 67 | - | Participants | p < 0.001 |
|  | Visuomotor mismatch responses on O1-2 vs P3-4 | Hierarchical Bootstrap | 133 | 65 | Electrodes | p < 0.001 |
|  | Visuomotor mismatch responses on O1-O2 vs C3-4 | Hierarchical Bootstrap | 133 | 57 | Electrodes | p < 0.001 |
|  | Visuomotor mismatch responses on O1-2 vs Fp1-2 | Hierarchical Bootstrap | 133 | 102 | Electrodes | p = 0.0140 |
| **Figure 3E** | Half peak latency on O1-2 vs C3-4 | Hierarchical Bootstrap | 133 | 57 | Electrodes | p = 0.1674 |
|  | Half peak latency on O1-2 vs P3-4 | Hierarchical Bootstrap | 133 | 65 | Electrodes | p = 0.4097 |
|  | Half peak latency on O1-2 vs Fp1-2 | Hierarchical Bootstrap | 133 | 102 | Electrodes | p = 0.0015 |
| **Figure 4A** | Normalized speed during visuomotor mismatch during early vs late trials | - | 54 | 54 | Participants | Black bar:  p < 0.05  Gray bar:  p > 0.05 |
| **Figure 4B** | Visuomotor mismatch responses on FP1-2 during early vs late trials | - | 46 | 46 | Participants | Black bar:  p < 0.05  Gray bar:  p > 0.05 |
| **Figure 4C** | Visuomotor mismatch responses on O1-2 during early vs late trials | - | 59 | 59 | Participants | Black bar:  p < 0.05  Gray bar:  p > 0.05 |
| **Figure 4D** | Comparison of visuomotor mismatch response ratios between early and late trials at O1-2 and Fp1-2 | Bootstrap | 59 | 46 | Participants | p = 0.0440 |
| **Figure 5A** | Visuomotor mismatch vs visual response on Fp1-2 | - | 59 | 29 | Participants | - |
|  | Visuomotor mismatch vs visual response on C3-4 | - | 30 | 30 | Participants | - |
|  | Visuomotor mismatch vs visual response on P3-4 | - | 35 | 31 | Participants | - |
|  | Visuomotor mismatch vs visual response on O1-2 | - | 75 | 29 | Participants | - |
| **Figure 5B** | Comparison of response power for visual and visuomotor mismatch stimulus on Fp1-2 | Bootstrap | 59 | 29 | Participants | p = 0.0140 |
|  | Comparison of response power for visual and visuomotor mismatch stimulus on C3-4 | Bootstrap | 30 | 30 |  | p < 0.001 |
|  | Comparison of response power for visual and visuomotor mismatch stimulus on P3-4 | Bootstrap | 35 | 31 |  | p < 0.001 |
|  | Comparison of response power for visual and visuomotor mismatch stimulus on O1-2 | Bootstrap | 75 | 29 |  | p < 0.001 |
| **Figure 6B** | Standard, deviant response and resulting difference waveform. | - | 22 | - | Participants | - |
| **Figure 6C** | Standard, deviant response and resulting difference waveform. | - | 22 | - | Participants | - |
| **Figure 6D** | Comparison of visuomotor mismatch, oddball mismatch and playback halt response. | - | Visuomotor mismatch (75), oddball mismatch (22), playback halt (42) | - | Participants | - |
| **Figure 6E** | Power of visuomotor mismatch vs oddball mismatch response | Bootstrap | 75 | 22 | Participants | p < 0.001 |
|  | Power of visuomotor mismatch vs playback halt response | Bootstrap | 75 | 42 | Participants | p < 0.001 |
|  | Power of oddball mismatch vs playback halt response | Bootstrap | 22 | 42 | Participants | p = 0.0196 |
| **Figure 1-figure supplement 2F** | Maximum amplitudes during visuomotor mismatch trials | - | 12976 | - | Trials | - |
| **Figure 1-figure supplement 3** | Visual response vs 0 on Fp1 | Bootstrap | 22 | - | Participants | p = 0.8794 |
|  | Visual response vs 0 on Fp2 | Bootstrap | 22 | - | Participants | p = 0.8090 |
|  | Visual response vs 0 on C3 | Bootstrap | 27 | - | Participants | p = 0.7820 |
|  | Visual response vs 0 on C4 | Bootstrap | 29 | - | Participants | p = 0.4476 |
|  | Visual response vs 0 on P3 | Bootstrap | 29 | - | Participants | p < 0.001 |
|  | Visual response vs 0 on P4 | Bootstrap | 30 | - | Participants | p = 0.0046 |
|  | Visual response vs 0 on O1 | Bootstrap | 25 | - | Participants | p < 0.001 |
|  | Visual response vs 0 on O2 | Bootstrap | 25 | - | Participants | p < 0.001 |
|  | Visual responses on O1-2 vs P3-4 | Hierarchical Bootstrap | 43 | 51 | Electrodes | p < 0.001 |
|  | Visual responses on O1-O2 vs C3-4 | Hierarchical Bootstrap | 43 | 48 | Electrodes | p < 0.001 |
|  | Visual responses on O1-2 vs Fp1-2 | Hierarchical Bootstrap | 43 | 29 | Electrodes | p < 0.001 |
| **Figure 2-figure supplement 1A** | Visuomotor mismatch responses | - | 14 | - | Participants | - |
| **Figure 2-figure supplement 1B** | 6DOF playback halt responses | - | 14 | - | Participants | - |
| **Figure 2-figure supplement 1C** | Comparison of visuomotor mismatch responses vs 6DOF playback halt responses | Bootstrap | 14 | 14 | Participants | p = 0.0048 |
| **Figure 2-figure supplement 1D** | 6DOF vs 4DOF playback halt responses | - | 16 | 42 | Participants | - |
| **Figure 2-figure supplement 1E** | Comparison of 6DOF vs 4DOF playback halt responses | Bootstrap | 16 | 42 | Participants | p = 0.2637 |
| **Figure 2-figure supplement 2A** | Visuomotor mismatch responses | - | 39 | - | Participants | - |
| **Figure 2-figure supplement 2B** | Playback halt responses | - | 39 | - | Participants | - |
| **Figure 2-figure supplement 2C** | Visuomotor mismatch vs playback halt responses | Bootstrap | 39 | 39 | Participants | p <0.001 |
| **Figure 2-figure supplement 3A** | Playback halt responses | - | 9 | - | Participants | - |
| **Figure 2-figure supplement 3B** | Visuomotor mismatch responses | - | 9 | - | Participants | - |
| **Figure 2-figure supplement 3C** | Comparison of playback halt vs visuomotor mismatch responses | Bootstrap | 9 | 9 | Participants | p = 0.0002 |
| **Figure 2-figure supplement 3D** | Strength of playback halt and visuomotor mismatch responses through the session | - | 9 | - | Participants | - |
| **Figure 2-figure supplement 3E** | Strength of visuomotor mismatch and playback halt responses through the session | - | 23 | - | Participants | - |
| **Figure 2-figure supplement 4A** | Horizontal eye position during visuomotor mismatch | - | 8 | - | Participants | Black bar:  p < 0.05  Gray bar:  p > 0.05 |
| **Figure 2-figure supplement 4B** | Vertical eye position during visuomotor mismatch | - | 8 | - | Participants | Black bar:  p < 0.05  Gray bar:  p > 0.05 |
| **Figure 2-figure supplement 4C** | Eye-movement speed during visuomotor mismatch | - | 8 | - | Participants | Black bar:  p < 0.05  Gray bar:  p > 0.05 |
| **Figure 2-figure supplement 4D** | Eye blinks during visuomotor mismatch | - | 8 | - | Participants | Black bar:  p < 0.05  Gray bar:  p > 0.05 |
| **Figure 2-figure supplement 4E** | Visuomotor mismatch responses | - | 75 | - | Participants | Black bar:  p < 0.05  Gray bar:  p > 0.05 |
| **Figure 2-figure supplement 5A** | Spectral power changes at electrodes Fp1-2 following visuomotor mismatch | - | 59 | - | Participants | - |
|  | Spectral power changes at electrodes C3-4 following visuomotor mismatch | - | 30 | - | Participants | - |
|  | Spectral power changes at electrodes P3-4 following visuomotor mismatch | - | 35 | - | Participants | - |
|  | Spectral power changes at electrodes O1-2 following visuomotor mismatch | - | 75 | - | Participants | - |
| **Figure 2-figure supplement 5B** | Spectral power changes at electrodes Fp1-2 following playback halt | - | 37 | - | Participants | - |
|  | Spectral power changes at electrodes C3-4 following playback halt | - | 31 | - | Participants | - |
|  | Spectral power changes at electrodes P3-4 following playback halt | - | 31 | - | Participants | - |
|  | Spectral power changes at electrodes O1-2 following playback halt | - | 42 | - | Participants | - |
| **Figure 2-figure supplement 6A** | ITPC across trials for electrodes Fp1-2 following visuomotor mismatch | - | 59 | - | Participants | - |
|  | ITPC across trials for electrodes C3-4 following visuomotor mismatch | - | 30 | - | Participants | - |
|  | ITPC across trials for electrodes P3-4 following visuomotor mismatch | - | 35 | - | Participants | - |
|  | ITPC across trials for electrodes O1-2 following visuomotor mismatch | - | 75 | - | Participants | - |
| **Figure 2-figure supplement 6B** | ITPC across trials for electrodes Fp1-2 following playback halt | - | 37 | - | Participants | - |
|  | ITPC across trials for electrodes C3-4 following playback halt | - | 31 | - | Participants | - |
|  | ITPC across trials for electrodes P3-4 following playback halt | - | 31 | - | Participants | - |
|  | ITPC across trials for electrodes O1-2 following playback halt | - | 42 | - | Participants | - |
| **Figure 6-figure supplement 1B** | Standard, deviant response and resulting difference waveform. | - | 9 | - | Participants | - |
| **Figure 6-figure supplement 1C** | Standard, deviant response and resulting difference waveform. | - | 9 | - | Participants | - |
| **Figure 6-figure supplement 1D** | Comparison of visuomotor mismatch, oddball mismatch and playback halt response. | - | Visuomotor mismatch (46), oddball mismatch (9), playback halt (11) |  | Participants | - |
| **Figure 6-figure supplement 1E** | Power of visuomotor mismatch vs oddball mismatch response | Bootstrap | 46 | 9 | Participants | p <0.001 |
|  | Power of visuomotor mismatch vs playback halt response | Bootstrap | 46 | 11 | Participants | p <0.001 |
|  | Power of oddball mismatch vs playback halt response | Bootstrap | 9 | 11 | Participants | p = 0.0404 |
| **Figure 6-figure supplement 2A** | Standard, deviant response and resulting difference waveform. | - | 9 | - | Participants | - |
| **Figure 6-figure supplement 2B** | Standard, deviant response and resulting difference waveform. | - | 9 | - | Participants | - |
| **Figure 6-figure supplement 2C** | Comparison of visuomotor mismatch, oddball mismatch and playback halt response. | - | Visuomotor mismatch (46), oddball mismatch (9), playback halt (11) | - | Participants | - |
| **Figure 6-figure supplement 2D** | Power of visuomotor mismatch vs oddball mismatch response | Bootstrap | 46 | 9 | Participants | p <0.001 |
|  | Power of visuomotor mismatch vs playback halt response | Bootstrap | 46 | 11 | Participants | p <0.001 |
|  | Power of oddball mismatch vs playback halt response | Bootstrap | 9 | 11 | Participants | p = 0.1886 |
