# Supplementary material for: Visuomotor mismatch EEG responses over occipital cortex of freely moving human subjects
Source: eLife. 2026 Aug 20;14:RP108941. doi: 10.7554/eLife.108941 (PMC13493123; doi:10.7554/eLife.108941)
Supplement: Supplementary file 3 [file elife-108941-supp2.docx]

| Paradigm | Session | Fp1-2 | C3-4 | P3-4 | O1-2 |
| --- | --- | --- | --- | --- | --- |
| Visual | Walking | 764/1610, **47.4%** | 428/1536, **27.9%** | 389/1536, **25.9%** | 597/1610, **37.1%** |
| Visual | Sitting | 379/1541, **24.6%** | 15/1455, **1.0%** | 14/1455, **1.0%** | 24/1541, **1.6%** |
| Visuomotor | Closed loop (walking) | 971/2200, **44.1%** | 318/1044, **30.5%** | 211/1044, **20.2%** | 478/2200, **21.7%** |
| Visuomotor | Open loop (sitting) | 241/1052, **22.9%** | 1/785, **0.1%** | 0/785, **0%** | 4/1052, **0.4%** |
