# Supplementary material for: Visuomotor mismatch EEG responses over occipital cortex of freely moving human subjects
Source: eLife. 2026 Aug 20;14:RP108941. doi: 10.7554/eLife.108941 (PMC13493123; doi:10.7554/eLife.108941)
Supplement: Peer review history

# Editor report: eLife Assessment

**DOI:** 10.7554/eLife.108941.4.sa0

**Authors:** Peter Kok

**Affiliations:** University College London United Kingdom

**Keywords:** Solid, Valuable

This **valuable** study demonstrates that self-motion strongly affects neural responses to visual stimuli, comparing humans moving through a virtual environment to passive viewing. The evidence for visuomotor mismatch responses is **solid**, although the interpretation in terms of prediction remains somewhat preliminary. This study bridges human and rodent studies on the role of prediction in sensory processing, and is therefore expected to be of interest to a large community of neuroscientists.

# Referee report: Reviewer #1 (Public review)

**DOI:** 10.7554/eLife.108941.4.sa1

In this paper, Solyga, Zelechowski & Keller study human visuomotor mismatch responses as an alternative instantiation of prediction errors to classic oddball paradigms. Using VR, they created a condition in which participants were moving around thereby creating a visuomotor coupling between physical movement and visual flow. To attempt to isolate the contribution of specifically movement-related predictions in this condition, they contrasted it to a condition in which participants were seated and rewatching their movement trajectory during the 'active' condition. Visuomotor mismatches were created by temporarily decoupling movement and visual experience by halting the VR display as participants continued to move.

The core finding of the paper is that participants exhibit a positively-valenced response to the visuomotor decoupling in the active but not in the passive condition. Since walking speed only insignificantly slows down following decoupling events in the active conditions, the authors argue that this difference cannot be accounted for by "changes in participants' behavior or to simple visual offset responses" with the latter being equal across both conditions. The following reinstatement of the coupling in turn does not differ between the two conditions. The authors additionally show that this mismatch response differs from visual onset responses elicited by checkerboard inversions and that it's "qualitatively" stronger than more commonly studied auditory oddball mismatch responses.

The design with its focus on ecological validity is impressive, well-rationalized and the results are well illustrated. I additionally appreciate the control analyses with regards to changes in walking speed and playback DOF and, now added, additional participants who experience the passive condition before the active.

My main question in round 1 regarded the isolation of visuomotor mismatch. Although the comparison with a seated control seems like a very sensible way to control for simple visual responses, there seem to be more differences than just a break in visuomotor coupling between the conditions. I therefore wonder whether the reduced offset response in the seated condition may be, in part, explained differently. For example, given that participants always conduct the active condition before rewatching their movement in the seated condition, it seemed likely that there is a component of learning across the session that flow will sometimes be halted. This is confirmed with the analyses. The explanation that there is a visuomotor component here is given further weight by their conduction of an additional group of participants who perform the conditions in the reverse order, so this has strengthened the manuscript considerably. However, it does of course remain an imperfect control because the visual stimulus is now different between the conditions for these participants. It's the best that can be achieved with this type of paradigm though and of course it yields a great deal of ecological validity.

I was also wondering whether the authors may consider the findings in frontal electrodes more closely given that the title of the paper focuses on a specifically occipital effect. Their further analyses have confirmed that there are likely interesting frontal effects. From a theoretical point of view, the spatial dissociation in adaptation effects, which were stronger in frontal and weaker in occipital areas, seems interesting and perhaps worth discussing, especially given the interpretation that "mismatch processing may initially arise in sensory visual areas before engaging higher-order frontal regions." How come the frontal decrease in responses is not accompanied by an analogous decrease in its supposed occipital source? Could these two responses reflect different kinds of prediction error signals (i.e. objective vs subjective)?

I remain concerned that the authors fight too defensively that they have absolutely isolated visuomotor prediction mechanisms with this paradigm. It's a nice, informative study, but it seems odd to argue there are no other possible explanations. One picks a design to optimize some features, but they will always come at some cost to others. Prioritising ecological validity, which is a justifiable aim, necessarily usually weakens some control over confounds.

To outline my reasoning fully: My concerns wrt generic influences of action on perception are reflected in Fig 1. The P1 is smaller when walking than sitting. It seems likely that the mismatch response reflects something about extrapolation or prediction, because it is larger when walking. However, it's not necessarily sensorimotor prediction. Even if you remove action from the equation, the flow can be extrapolated or predicted most of the time in a way it cannot so well when the video is halted. Of course, the sitting condition somewhat controls for it, but when it came second the visual flow disruptions were more predictable here. A reduction in effects over time is indeed confirmed with their analyses. They now have conducted a study with the conditions in the reverse order and they find the same thing. But of course, this necessitates non-identical visual flow because the sitting condition is playing the previous participant's flow. So it is likely that across all of these comparisons, it is the visuomotor mismatch that is especially salient. It's just that each comparison is a bit messy/confounded. It would strengthen the manuscript if there were some consideration given to the other processes likely at play here.

As a more minor point in response to our previous review, whether particular accounts represent an 'orthodox' view at present does not determine whether they raise logical issues in need of consideration. The authors may have missed that the papers in question consider mechanisms underlying the attenuation of particular pieces of information *from perception*. Not perceptual processing. We have one percept at any one moment in time and must understand how different population types synergistically generate that percept.

Similarly, a little strange is the way in which the authors aggressively defend the position that self-generated motion is 'the strongest' type of prediction. Sure, we probably experience the effects of our actions more often than ambulances. But what about objects obeying laws of gravity or others' faces being structured and moving in systematic ways? It is hard to quantify, such that presumably many scientists would be skeptical of such a claim, and it is not needed logically to justify the importance of examining mechanisms enabling action to shape perceptual processing. I'd assume it better to fight the battles you need to (and can) fight, such that the robust claims carry more weight.

Comments on latest version.

Nice to see the added extra analyses. Can't see any more will be achieved via further rounds and happy with the summary to stand as is.

# Referee report: Reviewer #2 (Public review)

**DOI:** 10.7554/eLife.108941.4.sa2

Summary:

This study investigates whether visuomotor mismatch responses can be detected in humans. By adapting paradigms from rodent studies, the authors report EEG evidence of mismatch responses during visuomotor conditions and compare them to visual-only stimulation and mismatch responses in other modalities.

Strengths:

- Authors use a creative experimental design to elicit visuomotor mismatch responses in humans.

- The study provides an initial dataset and analytical framework that could support future research on human visuomotor prediction errors.

Weaknesses:

- Methodological issues (e.g., volume conduction) make it difficult to confidently attribute the observed mismatch responses to activity in visual cortical regions. This could be alleviated by increasing the number of channels.

The authors successfully demonstrate that visuomotor mismatch paradigms can, in principle, be applied in human EEG. This approach provides a translational bridge between rodent and human work on predictive processing.

Comments on latest version.

The authors added a brief discussion paragraph which addresses my previous comment.

# Referee report: Reviewer #3 (Public review)

**DOI:** 10.7554/eLife.108941.4.sa3

Solyga, Zelechowski, and Keller present a concise report of an innovative study demonstrating clear visuomotor mismatch responses in ambulating humans, using a mobile EEG setup and virtual reality. Human subjects walked around a virtual corridor while EEGs were recorded. Occasionally, motion and visual flow were uncoupled, and this evoked a mismatch response that was strongest in occipitally placed electrodes and had a considerable signal to noise ratio. It was robust across participants and could not be explained by the visual stimulus alone.

This is an important extension of their prior work in mice and represents an elegant translation of those previous findings to humans, where future work can inform theories of e.g. psychiatric diseases that are believed to involve disordered predictive processing. For the most part, the authors are appropriately circumspect in their interpretations and discussions of the implications. The paper in its current form represents an important addition to the literature.

The authors have included analyses of the auditory mismatch using temporal electrodes, referenced to Cz (and therefore should exhibit a mismatch positivity). This added data clearly and convincingly shows that the sensorimotor mismatch is, indeed, stronger than the passive auditory MMN.

Comments on latest version:

The authors added useful points to the discussion and also included time frequency analyses to the paper formally, which strengthens the translational potential, in addition to the bolstering their claims slightly.

# Author comment: Author response

**DOI:** 10.7554/eLife.108941.4.sa4

**Authors:** Magdalena Solyga, Marek Zelechowski, Georg B Keller

**Affiliations:** Friedrich Miescher Institute Basel Switzerland; Center for medical Image Analysis and Navigation, Department of Biomedical Engineering, University of Basel Basel Switzerland; Friedrich Miescher Institute Basel Switzerland

The following is the authors’ response to the previous reviews

We thank you for the time you took to review our work and for your feedback! The main changes to the manuscript are:

We added a paragraph to the Discussion addressing differences in visuomotor mismatch responses recorded over frontal and occipital electrodes, and their possible interpretation.

We added time-frequency power and phase-locking analysis as supplementary figures to the manuscript.

We added a statement in the Discussion emphasizing the importance of performing these experiments with denser EEG channel coverage.

**Public Reviews:**

**Reviewer #1 (Public review):**

In this paper, Solyga, Zelechowski & Keller study human visuomotor mismatch responses as an alternative instantiation of prediction errors to classic oddball paradigms. Using VR, they created a condition in which participants were moving around thereby creating a visuomotor coupling between physical movement and visual flow. To attempt to isolate the contribution of specifically movement-related predictions in this condition, they contrasted it to a condition in which participants were seated and rewatching their movement trajectory during the 'active' condition. Visuomotor mismatches were created by temporarily decoupling movement and visual experience by halting the VR display as participants continued to move.

The core finding of the paper is that participants exhibit a positively-valenced response to the visuomotor decoupling in the active but not in the passive condition. Since walking speed only insignificantly slows down following decoupling events in the active conditions, the authors argue that this difference can not be accounted for by "changes in participants' behavior or to simple visual offset responses" with the latter being equal across both conditions. The following reinstatement of the coupling in turn does not differ between the two conditions. The authors additionally show that this mismatch response differs from visual onset responses elicited by checkerboard inversions and that it's "qualitatively" stronger than more commonly studied auditory oddball mismatch responses.

The design with its focus on ecological validity is impressive, well-rationalized and the results are well illustrated. I additionally appreciate the control analyses with regards to changes in walking speed and playback DOF and, now added, additional participants who experience the passive condition before the active. I have a couple of questions/comments.

My main question in round 1 regarded the isolation of visuomotor mismatch. Although the comparison with a seated control seems like a very sensible way to control for simple visual responses, there seem to be more differences than just a break in visuomotor coupling between the conditions. I therefore wonder whether the reduced offset response in the seated condition may be, in part, explained differently. For example, given that participants always conduct the active condition before rewatching their movement in the seated condition, it seemed likely that there is a component of learning across the session that flow will sometimes be halted. This is confirmed with the analyses. The explanation that there is a visuomotor component here is given further weight by their conduction of an additional group of participants who perform the conditions in the reverse order, so this has strengthened the manuscript considerably. However, it does of course remain an imperfect control because the visual stimulus is now different between the conditions for these participants. It's the best that can be achieved with this type of paradigm though and of course it yields a great deal of ecological validity.

The reviewer is correct. But one should keep in mind that our result here stands in the context of a considerable amount of work on mouse cortex investigating responses to very similar visuomotor mismatches. There we can we have much additional evidence to argue that the cortical response to a visuomotor mismatch is a prediction error. We would argue, it is the best one can do in human experiments.

I was also wondering whether the authors may consider the findings in frontal electrodes more closely given that the title of the paper focuses on a specifically occipital effect. Their further analyses have confirmed that there are likely interesting frontal effects. From a theoretical point of view, the spatial dissociation in adaptation effects, which were stronger in frontal and weaker in occipital areas, seems interesting and perhaps worth discussing, especially given the interpretation that "mismatch processing may initially arise in sensory visual areas before engaging higher-order frontal regions." How come the frontal decrease in responses is not accompanied by an analogous decrease in its supposed occipital source? Could these two responses reflect different kinds of prediction error signals (i.e. objective vs subjective)?

We have added a paragraph to the Discussion addressing the differences between signals recorded over frontal and occipital electrodes, as suggested.

I remain concerned that the authors fight too defensively that they have absolutely isolated visuomotor prediction mechanisms with this paradigm. It's a nice, informative study, but it seems odd to argue there are no other possible explanations. One picks a design to optimize some features but they will always come at some cost to others. Prioritising ecological validity, which is a justifiable aim, necessarily usually weakens some control over confounds.

We are not sure what the reviewer is referring to here. We certainly do not think (or are aware of having argued) that a visuomotor prediction error is the only possibly interpretation of the response. In the last paragraph of our response to the reviewers point 3 in the last revision, we explicitly discuss that the interpretation of the responses as a prediction error is only one possible interpretation. Our argument is that it is the most likely given the evidence.

To outline my reasoning fully: My concerns wrt generic influences of action on perception are reflected in Fig 1. The P1 is smaller when walking than sitting. It seems likely that the mismatch response reflects something about extrapolation or prediction, because it is larger when walking. However, it's not necessarily sensorimotor prediction. Even if you remove action from the equation, the flow can be extrapolated or predicted most of the time in a way it cannot so well when the video is halted. Of course the sitting condition somewhat controls for it, but when it came second the visual flow disruptions were more predictable here. A reduction in effects over time is indeed confirmed with their analyses. They now have conducted a study with the conditions in the reverse order and they find the same thing. But of course this necessitates non-identical visual flow because the sitting condition is playing the previous participant's flow. So it is likely that across all of these comparisons, it is the visuomotor mismatch that is especially salient. It's just that each comparison is a bit messy/confounded. It would strengthen the manuscript if there were some consideration given to the other processes likely at play here.

We would be happy to add additional considerations to other processes. If the reviewer has anything specific in mind, we can add that, but it would need to be somewhat concrete with some theoretical basis. We share the reviewer’s intuition, but unless this can be formalized to the point of being experimentally testable, we do not see any value in discussing it in the manuscript.

Regarding the reason for a difference in visual responses in walking vs sitting state is, this is not entirely clear to us. Predictive processing would provide one possible explanation. Assuming the precision weighting of predictions is higher during walking, the sudden appearance of a visual stimulus might lead to stronger stimulus history prediction errors than when just sitting. But this is rather speculative.

As a more minor point in response to our previous review, whether particular accounts represent an 'orthodox' view at present does not determine whether they raise logical issues in need of consideration. The authors may have missed that the papers in question consider mechanisms underlying the attenuation of particular pieces of information ‘from perception’. Not perceptual processing. We have one percept at any one moment in time and must understand how different population types synergistically generate that percept.

Please excuse, the reviewer is correct, the orthodoxy of an idea is not relevant. For dubious reasons, we chose to euphemize what we actually meant to say here. With regards to circuit implementations of predictive processing (we cannot and do not intend to speak to interpretations of predictive processing that relate to conscious perception much of V1 activity is likely not consciously perceived – we assume this is what the reviewer is referring to by “we have one percept”) – the reviewers interpretation was not unorthodox, but rather incorrect (which is what we should have said). The statement that “the brain predictively ‘cancels’ expected action outcomes from perception” is incorrect in the context of sensory processing – based on both theoretical models of predictive processing, and more importantly physiological evidence. If the point was only in regards to conscious perception, we also suspect the statement is wrong, but even if it were correct, don’t see how it pertains to our work.

Similarly a little strange is the way in which the authors aggressively defend the position that self-generated motion is 'the strongest' type of prediction. Sure, we probably experience the effects of our actions more often than ambulances. But what about objects obeying laws of gravity or others' faces being structured and moving in systematic ways? It is hard to quantify, such that presumably many scientists would be skeptical of such a claim, and it is not needed logically to justify the importance of examining mechanisms enabling action to shape perceptual processing. I'd assume it better to fight the battles you need to (and can) fight, such that the robust claims carry more weight.

We believe it is absolutely essential for the progress of the field that we start to emphasize the differences between something that is “predictable in principle” and “predicted by the brain”. There is likely indeed a hierarchy of predictability that looks something like this:

(1) Sensorimotor coupling

(2) Laws of physics

(3) Behavior of other living things

(4) Artificial, human-made statistical relationships

Almost all published experiments are based on the fourth type of prediction. Indeed, why not use physics simulations instead of oddballs and MMN? We absolutely should! But the field tends to revert to artificial couplings. As a direct consequence of this, the number of papers appearing recently (from both human and mouse fields), that are built on the following premise:

(1) Expose an animal or human to an artificial coupling between A and B (e.g. an oddball, or a global oddball, or any of a myriad other constructions).

(2) Probe for prediction error responses to the violation of the artificial coupling.

(3) Find no prediction error responses and conclude predictive processing is wrong.

Is utterly baffling. The fallacy here is of course the assumption that if something is predictable in principle, the brain must predict it. Thus, we are, and will continue to be strong on this point, and we think it is essential that we – as a field – are.

Hope these comments are helpful.

**Reviewer #2 (Public review):**

Summary:

This study investigates whether visuomotor mismatch responses can be detected in humans. By adapting paradigms from rodent studies, the authors report EEG evidence of mismatch responses during visuomotor conditions and compare them to visual-only stimulation and mismatch responses in other modalities.

Strengths:

Authors use a creative experimental design to elicit visuomotor mismatch responses in humans.

The study provides an initial dataset and analytical framework that could support future research on human visuomotor prediction errors.

Weaknesses:

Methodological issues (e.g., volume conduction) make it difficult to confidently attribute the observed mismatch responses to activity in visual cortical regions. This could be alleviated by increasing the number of channels.

We have added a discussion of this.

The authors successfully demonstrate that visuomotor mismatch paradigms can, in principle, be applied in human EEG. This approach provides a translational bridge between rodent and human work on predictive processing.

**Reviewer #3 (Public review):**

Solyga, Zelechowski, and Keller present a concise report of an innovative study demonstrating clear visuomotor mismatch responses in ambulating humans, using a mobile EEG setup and virtual reality. Human subjects walked around a virtual corridor while EEGs were recorded. Occasionally, motion and visual flow were uncoupled, and this evoked a mismatch response that was strongest in occipitally placed electrodes and had a considerable signal to noise ratio. It was robust across participants and could not be explained by the visual stimulus alone.

This is an important extension of their prior work in mice, and represents an elegant translation of those previous findings to humans, where future work can inform theories of e.g. psychiatric diseases that are believed to involve disordered predictive processing. For the most part, the authors are appropriately circumspect in their interpretations and discussions of the implications. The paper in its current form represents an important addition to the literature.

The authors have included analyses of the auditory mismatch using temporal electrodes, referenced to Cz (and therefore should exhibit a mismatch positivity). This added data clearly and convincingly shows that the sensorimotor mismatch is, indeed, stronger than the passive auditory MMN.

The reference electrode placed at Cz makes it is difficult to interpret relative differences between frontal and occipital electrode responses, as the occipital electrodes are placed farther away from the Cz reference than the frontal electrodes. Similarly, signal occuring cortically near the Cz reference might only appear as though it is occipitally distributed in this montage. It is common in EEG research to remontage the data to an averaged common reference in order to better interpret the scalp distributions. As the electrode coverage was sparse for some subjects, this could be challenging, and this reviewer does not feel that it is necessary to do this analysis step, or even to drastically rewrite the body of the paper. We only request that some discussion, however brief, is included in the discussion section or the methods that recommend more dense electrode coverage in the future to better interpret scalp distributions and potential meso-scale sources.

We have added a discussion of this as suggested.

This is just a suggestion. The authors are encouraged to analyse (and report) time-frequency power and phase locking for these mismatch responses, as is common in much of the literature (see Roach et al 2008 Schizophrenia Bulletin). This is not to say that doing so will yield insights into oscillations per se, but converting the data to the time-frequency domain provides another perspective that has some advantages. fosters translations to rodent models, as ERP peaks do not map well between species, but e.g. delta-theta power does (see Lee et al 2018 Neuropsychopharmacology; Javitt et all 2018 Schizophrenia research; Gallimore et al 2023 Cereb Ctx). Further, ERP peaks can be influenced by the actual neuroanatomy of an individual (especially for quantifying V1 responses). Time frequency analyses may aid in interpreting the "early negative deflection with a peak latency of 48 ms " finding as well. As it stands, the report is complete, and it would be acceptable if the authors chose to save this type of analysis for a future publication.

We have added this as suggested.

**Recommendations for the authors:**

**Reviewer #2 (Recommendations for the authors):**

The authors have addressed most of my concerns by providing additional analyses, partly based on new data. The volume conduction issue is partly addressed based on the result showing latency differences, however to confidently assign responses to visual regions, one would need to perform recordings with a larger number of electrodes, sufficient to perform source localization. Nevertheless, the manuscript is now more solid than the previous version.

We have now added this point to the Discussion.

**Reviewer #3 (Recommendations for the authors):**

The reviewer appreciates that the authors have carried out time-frequency analyses, and are ok with them leaving this out of this paper.

We have now added this to the manuscript.

Finally, in response to the participant quote "are you printing this? hi mom!" - this reviewer concedes that it does not significantly detract from the report, and, in the interest of amusement and joy, would abide its reinstatement.

We greatly appreciate the reviewers entertaining our attempts at humor but will leave it out as originally suggested.
